# Three-dimensional rotational angiography improves mechanical thrombectomy recanalization rate for acute ischaemic stroke due to middle cerebral artery M2 segment occlusions

**DOI:** 10.1177/15910199221145745

**Published:** 2022-12-18

**Authors:** Andrea Rosi, Gianmarco Bernava, Jeremy Hofmeister, Madruzzato Nicolò, José Boto, Hasan Yilmaz, Philippe Reymond, Olivier Brina, Michel Muster, Emmanuel Carrera, Karl-Olof Lövblad, Paolo Machi

**Affiliations:** 1 Division of Diagnostic and Interventional Neuroradiology, Department of Diagnostics, 27230 Geneva University Hospitals, Geneva, Switzerland; 2 Brain Endovascular Therapeutics Research and Development Laboratory, Radiology and Medical Informatics, 27212 University of Geneva, Geneva, Switzerland; 3 Neuroradiology, Verona University Hospital, Verona, Italy; 4 Departement of Surgery, 27230 Geneva University Hospitals, Geneva, Switzerland; 5 Division of Neurology, Department of Neurosciences, 27230 Geneva University Hospitals, Geneva, Switzerland

**Keywords:** Stroke, thrombectomy, M2, MeVO, 3D-RA

## Abstract

**Background:**

Occlusions of the middle cerebral artery (MCA) M2 segments can be difficult to address with mechanical thrombectomy (MTB) using standard projections and this can affect the final recanalization. Three-dimensional rotational angiography (3D-RA) allows to obtain a 3D model of cerebral vessels in a few seconds and to determine the best two-dimensional (2D) projections to be selected to evaluate and treat cerebrovascular diseases, such as aneurysms or vascular malformations. We aimed to determine if 3D-RA could be applied also in MTB.

**Methods:**

A retrospective review of two patient cohorts treated during two time periods of 12 months before and after the introduction of 3D-RA use at our institution for MTB in M2 occlusions. Analyses were conducted to compare the two groups for procedural characteristics, such as timing, recanalization rate and complications and clinical outcome.

**Results:**

One hundred acute ischaemic stroke (AIS) patients (3D-RA group = 57; controls = 43) underwent MTB for an M2 occlusion during the two study periods. Recanalization rates were significantly higher in cases treated with 3D-RA. The mean 3D technique thrombectomy time was compared to that of non-3D cases (47 vs. 49 min, respectively).

**Conclusions:**

Our findings showed that 3D-RA is a useful tool to select specific working projections to AIS patients presenting an M2 occlusion by improving final recanalization compared to standard projections, without increasing the overall procedural time.

## Introduction

The optimal visualization of the occluded artery is pivotal to navigate thrombectomy devices over the clot to effectively perform mechanical thrombectomy (MTB). MTB is generally performed under the guidance of standard antero-posterior (AP) and latero-lateral (LL) digital subtracted angiography (DSA) projections, which are generally suitable to depict the occluded artery in the case of large vessel occlusion, as well as the vascular anatomy proximal to the occlusion. However, in cases of middle cerebral artery-medium vessel occlusion (MCA-MeVO),^1^ standard projections are often not suitable to provide guidance for an eventual MTB given the potential presence of other vessels hiding the occluded artery. In these cases, MTB is often conducted under the guidance of projections only poorly depicting the occlusion, despite several angiographic acquisitions, which could potentially result in a delayed or inefficacious occluded artery recanalization.^2^

Since 2018, we have performed three-dimensional rotational angiography (3D-RA) at our centre to select the most accurate two-dimensional (2D) angiographic projections to provide guidance to the MTB in cases of acute ischaemic stroke (AIS) due to an M2 occlusion. The rationale behind this approach is that 3D-RA favours the precise visualization of the occlusion and the vessel anatomy proximal to the occlusion, thus providing guidance for a rapid and effective MTB. To the best of our knowledge, the use of 3D-RA to guide MTB has not been previously reported so far.

In the present study, we conducted a retrospective evaluation of the clinical and anatomical results of a cohort of consecutive AIS patients presenting with an M2 occlusion treated at our institution by MTB and for which a 3D-RA was acquired during the procedure (3D-RA group). Results were compared with a similar cohort of patients presenting with an M2 occlusion treated during the same time period (12 months) before the introduction of 3D-RA use for MTB (control group) in order to evaluate whether a 3D-RA approach had an impact on MTB efficacy, more specifically on the racanalization rate.

## Methods

### Patients and procedures

#### 3D-RA group

This cohort included patients treated in the 12-month period (between July 2018 and August 2019) following the introduction of the 3D-RA approach for MTB in July 2018. 3D-RAs were acquired in three specific circumstances^1^: (1) at the beginning of the MTB when the pre-procedural CT- or MR angiography predicted the presence of an M2 occlusion (primary M2 occlusion); (2) in the presence of a distal embolus resulting from the fragmentation of a more proximal clot initially located in M1 or ICA (secondary M2 occlusion); and (3) in the presence of a distal embolus already present before the removal of a more proximal thrombus (concurrent M2 occlusion). In such cases, the 3D-RA was acquired after recanalization of the proximal occlusion.

#### Control group

This second cohort included patients treated during a 12-month period (between May 2017 and June 2018) preceding the introduction of the 3D-RA approach for MTB and presenting an occlusion pattern for which a 3D-RA would be acquired. MTB was mainly performed among these patients under the guidance of standard AP, LL and oblique digital subtracted angiography (DSA) projections.

#### Pre-procedural imaging selection

According to our institutional protocol, all AIS patients were evaluated upon hospital arrival by clinical assessment, non-contrast enhanced CT, CT perfusion, CT angiography and an automated CT perfusion software (RAPID software; iSchemaView, Menlo Park, CA, USA). The clinical criteria for an indication for MTB were an NIHSS ≥ 6 or an NIHSS < 6 but with a disabling clinical deficit (i.e. aphasia or hemiplegia). MTB has been offered to all patients presenting within 8 h from stroke onset, regardless of ischaemic core extent. For patients presenting between the ages of 8 and 24, MTB was offered to patients according to American Heart Association/American Stroke Association guidelines (ischaemic core extent at perfusion CT ≤70 ml).^3^ These selection criteria applied to both patients with intracranial occlusions of the internal carotid artery or the M1 segment of the MCA (who were treated for secondary or concomitant M2 occlusions during the same endovascular procedure) and patients with primary M2 occlusions.

#### 3D-RA protocol

**Figure 1.**
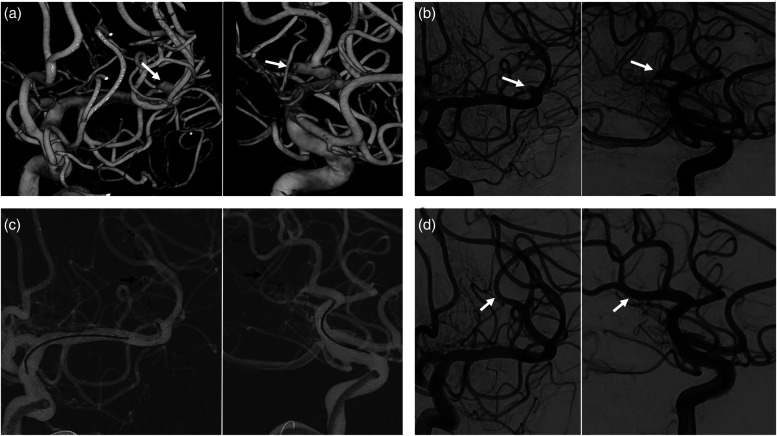
Illustrative case of the use of three-dimensional rotational angiography (3D-RA) in a left M2 occlusion. A patient presenting with a left M2 occlusion was treated by selecting the working projections on the base of 3D-RA. (a) Two 3D-RA views showing the occluded branch (white arrows); (b) their corresponding working projection views acquired with biplanar digital subtracted angiography (DSA); (c) the mechanical thrombectomy (MTB) done by deploying a stent retriever (STR) (black arrows) under the guidance of the roadmap derived from the working projection biplanar DSA; and (d) the re-perfused M2 branch after the thrombectomy. A flowchart including the initial and final standard antero-posterior (AP) and latero-lateral (LL) DSA projections of this case can be found in Supplementary material.

**Figure 2.**
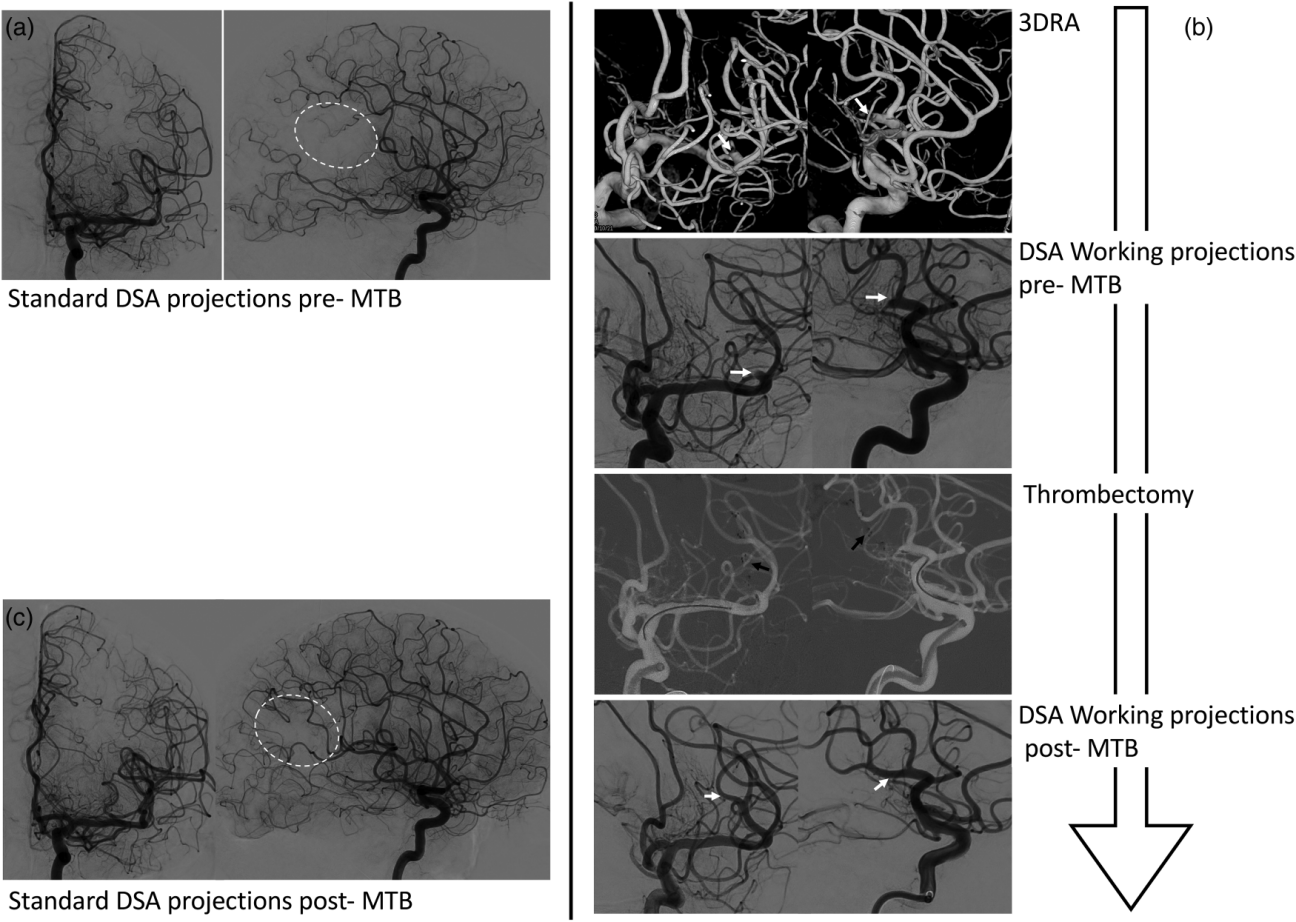
Illustrative case of the flowchart to use the three-dimensional rotational angiography (3D-RA)-derived projections compared to standard ones in a patient presenting an occlusion of M2 segment. (a) The standard digital subtracted angiography (DSA) projection views (antero-posterior (AP) and latero-lateral LL)) show the vascularization defect of the brain territory (white dotted line), but provides no information about the location of the occluded branch. (b). The proposed workflow shows from top to bottom: (1) two 3D-RA views showing the occluded branch (white arrows); (2) their corresponding DSA working projection views; (3) the mechanical thrombectomy (MTB) using a stent retriever (STR) (black arrows); and (4) the re-perfused M2 branch. (c) The standard DSA projection views showing the re-vascularization recovery of the M2 branch brain territory (white dotted line).

MTB procedures were performed using a biplane C-arm Allura Clarity FD20 (Philips Healthcare, Best, The Netherlands). The 3D-RA protocol used for MTB was the same as the one used at our institution for cerebral aneurysm evaluation. A volume of 18 ml of non-ionic contrast medium (Iopamiro 300 mg/ml, Bracco, Milan, Italy) was injected with an automated injector at a flow rate of 3 ml/s up to 52 bar/754 psi through the guiding or the intermediate (aspiration) catheter placed in the cervical segment of the concerned internal carotid artery. Acquired raw data were reconstructed using the Xtravision workstation (Philips Healthcare) to obtain 3D images subsequently analysed to select the working projections. In the 3D-RA group, 3D acquisitions were used to select the most appropriate 2D working projections showing the arterial anatomy upstream of the occlusion and up to the proximal edge of the clot. 2D angiography projections were acquired using a field of view of 15 cm to allow an optimal visualization of the occluded artery. An illustrative case of the selection of working projections based on 3D-RA to perform thrombectomy of an M2 occlusion is shown in Figures 1 and 2. A video containing another illustrative case, intended to show the step-by-step selection and utilization of working projections obtained with 3D-RA, has been added as Supplementary material.

#### MTB procedure

MTBs were performed with the patient under general anaesthesia. If general anaesthesia was contraindicated, the procedure was performed under conscious sedation. Eligible patients received intravenous recombinant tissue plasminogen activator (rtPA; Actilyse; Boehringer Ingelheim, Basel, Switzerland) prior to MTB. Procedures were performed by direct thromboaspiration^4^ or using a combined technique in which stent retriever (STR) thrombectomies were combined with distal aspiration performed with aspiration catheters placed in proximity of the occlusion point.^5^ In cases of combined technique, the STR was completely retrieved through the aspiration catheter outside the patient's body, while the aspiration catheter was maintained in proximity of the occlusion point or within the field of view in order to facilitate additional STR passes if needed. During STR retrievals, a continous negative pressure was generated inside the aspiration catheters using a vacuum system (Medela, Stryker, Kalamazoo, MI, USA). At the end of all procedures a cone beam CT (X-per CT; Allura Xper FD20; Philips Healthcare) was performed in order to rule out the presence of haemorrhagic complications.

The study was approved by the local ethics committee for research on human subjects, which waived the need for written informed consent.

### Assessment of variables

MTB variables (timing, number of MTB passes, target artery recanalization and occurrence of intraprocedural complications) and 3-month clinical outcome were retrospectively assessed by two raters with expertise in AIS treatment (PM and AR) based on patients’ records and intraprocedural DSA imaging.

In addition, in order to investigate whether the use of 3D involved a significant amount of additional time, the reviewers evaluated the timing of the various phases of the 3D approach.

For M2 occlusions MTB timing, raters evaluated the overall time interval between the first angiograms showing the M2 occlusion and the final post-treatment angiographic series for both groups. In cases of secondary or concomitant M2 occlusion, the time intervals of the 3D approach were calculated from the moment at which the M2 occlusion was detected, that is,. after the recanalization of the proximal occlusion. This was done in order make the timing of these cases compared to those of MTB for primary M2 occlusions.

The time spent between the first DSA angiogram showing the M2 occlusion and the acquisition of the 3D-RA and the time interval between the 3D-RA and the acquisition of the specific 2D working projections. The sum of these two time intervals represented the total time spent to acquire the specific projections since the M2 occlusion was detected.

Raters also reviewed periprocedural images (DSA and follow-up CT) to assess the number of MTB passes, the rate of target vessel recanalization, the occurrence of embolization in new vascular territories (ENT) and post-procedural intracranial haemorrhages.

Target vessel recanalization was assessed for the whole MCA territory according to the modified thrombolysis in cerebral infarction (mTICI) score^6, 7^ (Table 1), with an mTICI ≥2b defined as a good recanalization. Nevertheless, given the distality of the occlusions of our patient populations, the mTICI was not always appropriate to adjudicate a favourable recanalization as most patients presented an mTICI of 2b from the beginning of the procedure. Hence, to evaluate MTB efficacy in these patients, raters assessed whether the initial mTICI improved or not along the procedure (mTICI ‘shift’) (Table 1). For the same reason, the degree of the MCA-MeVO recanalization was also assessed according to the mTICI classification for MCA-MeVOs in the middle cerebral artery (MeVO-M-TICI)^1, 8^ (Table 1).

**Table 1.** Reperfusion scoring systems applied in the study.

Scoring system name	Scores	Description
mTICI score (overall MCA territory)^6, 7^	0	No reperfusion of the MCA territory.
	1	Minimal reperfusion of the MCA territory.
	2a	Partial filling <50% of the MCA territory.
	2b	Partial filling ≥50% of the MCA territory.
	2c	Near-complete reperfusion except for slow flow in one or two distal cortical vessels or the presence of minor distal emboli.
	3	Complete reperfusion of the MCA territory.
mTICI score shift (final vs. at 3D)	mTICI unchanged	Comparison between the mTICI score for the overall MCA territory at the beginning of the procedure and the mTICI score for the same territory at the end of the procedure.
	mTICI improved	
MeVO-M-TICI score^1, 8^	0	Scoring system identical to the standard TICI used for the overall MCA territory. The key difference with the conventional TICI score is that the ‘denominator’, which is only the affected territory downstream to the MeVO, is used as the comparator, rather than the entire MCA territory.
	1	
	2a	
	2b	
	2c	
	3	

This table includes the reperfusion scoring systems that were applied to assess the angiographic outcome of thrombectomies for the M2 occlusions of the patients included in the study.

MCA: middle cerebral artery; MeVO: medium vessel occlusion; MeVO-M-TICI: modified TICI classification for MeVOs in the middle cerebral artery; mTICI: modified thrombolysis in cerebral infarction.

The 3-month patient clinical outcome, evaluated according to the modified Rankin scale (mRS), was obtained from the patients’ clinical records or evaluated by telephone interview with the patient or family, if not available. A 3-month mRS ≤ 2 was considered to be a favourable clinical outcome.

### Statistical analyses

We compared the procedural timing and number of MTB passes between the 3D-RA and control groups using the independent samples t-test after performing a Levene's test for equality of variances. Equal variances were assumed only in the case of a non-significant result in Levene's test. We used a chi-square test for the comparison of categorical and ordinal variables between the two groups (final mTICI score, mTICI score shift, degree of recanalization of the target vessel, characteristics of residual occlusions, intraprocedural complications and mRS at 3 months). Moreover, we assessed the effect of 3D-RA on the mTICI score shift and the characteristics of residual occlusion using logistic regression. All statistical analyses were performed on SPSS Version 22, with a two-tailed 0.05 significance level.

## Results

Patients’ demographic and procedure variables are reported in Table 2.

**Table 2.** Patients’ demographic and procedure variables.

		Control group		3D-RA group	*P*-value
Number of patients		43/100 (43%)		57/100 (57%)	
Age [mean ± SD]		71 (±14) years		74 (±14) years	
Gender [females]		22/43 (51%)		31/57 (54.4%)	0.840
Primary MeVO		22/43 (51.2%)		34/57 (59.6%)	0.423
Thrombolysis (intravenous rtPA)		23/43 (53.5%)		33/57 (57.9%)	1
Time to recanalization^a^ [mean ± SD]		52,3 (± 36.5) min.		48,7 (± 29.9) min.	0.596
Time between first visualization of M2 occlusion and 3D acquisition (minutes) [mean (SD)]		NA		3.9 (± 2.4)	
MTB passes in the selected branch [median, IQR]		2 (1–4)		2 (1–4)	0.411
Final mTICI score (overall MCA territory)	0–1	0/43 (0%)	0/57 (0%)		
	2a	11/43 (25.6%)	0/57 (0%)		
	2b	14/43 (32.6%)	17/57 (29.8%)		<0.001
	2c	4/43 (9.3%)	9/57 (15.8%)		
	3	14/43 (32.6%)	31/57 (54.4%)		
mTICI score shift (final vs. at 3D)	mTICI unchanged	15/43 (34.9%)	4/57 (7%)		
	mTICI improved	28/43 (65.1%)	53/57 (93%)		0.001
Final MeVO-M-TICI score	1-2a	16/43 (37.2%)	0/57 (0%)		
	2b	12/42 (27.9%)	26/57 (45.6%)		<0.001
	2c-3	15 (34.9%)	31/57 (54.4%)		
MTB technique	Aspiration without STR	6/43 (14%)	5/57 (8.8.%)		
	Aspiration with STR	37/43 (86%)	52/57 (91.2%)		0.523
Complications	ENT Haemorrhage Total	1/43 (2.3%) 1/43 (2.3%) 2/43 (4.7%)	1/57 (1.8%) 3/57 (5.3%) 4/57 (7%)		0.697
Overall population mRS at 3 months	mRS 0–2 mRS 3–6	18/40 (45%) 22/40 (55%)	26/53 (49.1%) 27/53 (50.9%)		0.834
Primary M2 occlusions mRS at 3 months	mRS 0–2 mRS 3–6	10/22 (45.5%) 12/22 (54.5%)	19/31 (61.3%) 12/31 (38.7%)		0.253

^a^ Time interval between the first 2D angiograms showing the M2 occlusion and the final post-treatment angiographic series.

DSA: digital subtracted angiography; ENT: embolization to a new territory; IQR: interquartile range; mRS: modified Rankin scale; MeVO: medium vessel occlusion; MeVO-M-TICI: modified TICI classification for MCA-MeVOs in the middle cerebral artery; MTB: mechanical thrombectomy; mTICI: modified thrombolysis in cerebral infarction; NA: not applicable; rtPA: recombinant tissue plasminogen activator; SD: standard deviation; STR: stent retriever.

One hundred AIS patients underwent MTB at our institution for an M2 occlusion during the study period. Of these, 43 patients underwent MTB without the guidance of 3D-RA (control group), while the remaining 57 patients were treated using 3D-RA guidance (3D-RA group). In the overall study population, 56 patients (56%) were treated for primary M2 occlusions; these constituted the 59.6% (34/57) of the 3D-RA group and the 51.2% (22/43) of the control group.

All patients were treated under general anaesthesia according to our institutional protocol, except for four patients in the control group who were treated under conscious sedation due to cardio-respiratory contraindications to general anaesthesia. There was no difference between groups concerning the proportion of patients who received recombinant tissue plasminogen activator (rtPA) prior to MTB (*p* = 1.000), the initial mTICI score (*p* = 0.149) and the technique used for MTB, that is, direct thromboaspiration or combined approach (*p* = 0.523).

### Recanalization rate

**Figure 3.**
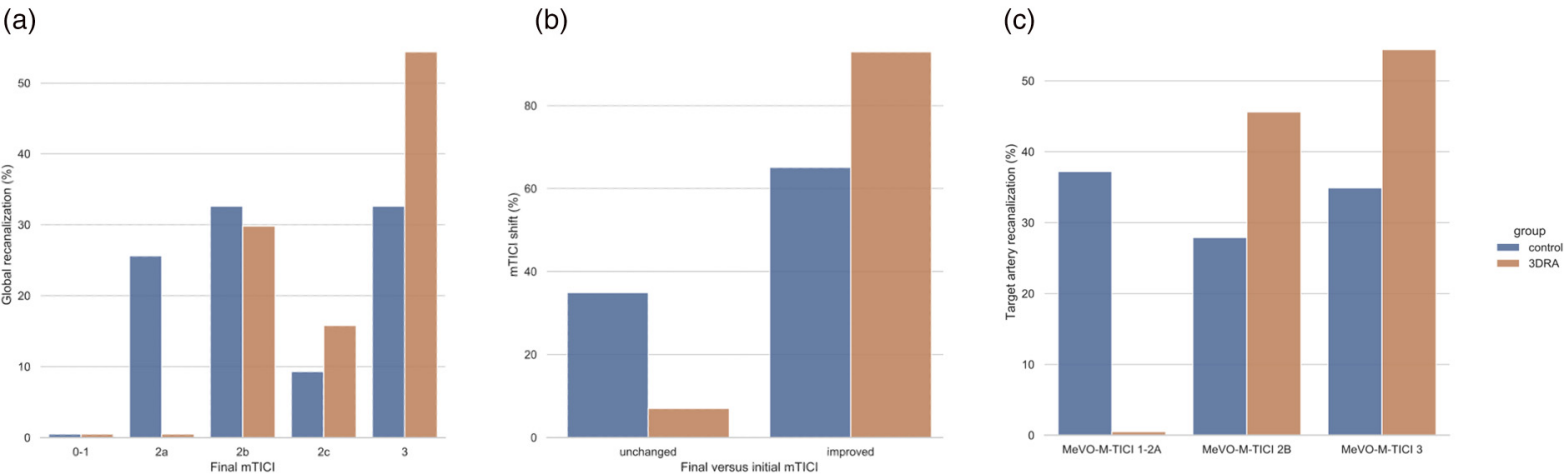
Comparison of recanalization rates. Histograms showing the better recanalization after endovascular treatment of M2 occlusion in the group of patients treated with 3D technique when compared with the control group, taking into account multiple angiographic outcomes. (a) Comparison of the percentages of patients with different degrees of recanalization according to the modified thrombolysis in cerebral infarction (mTICI) scale between the two groups under study. (b) Comparison of the percentages of patients in the two groups who showed an unchanged or improved mTICI at the end of endovascular treatment when compared with the start of M2 occlusion treatment. (c) Comparison of the percentages of patients with different degrees of recanalization according to the medium vessel occlusion (MeVO)-M-TICI scale between the two study groups.

All patients (57/57 [100%]) in the 3D-RA group had a good reperfusion outcome (mTICI ≥2b) compared to 32/43 (74.4%) in the control group (*p* < 0.001) (Figure 3(a)). Logistic regression also showed that the odds of improvement of the mTICI for the overall MCA territory at the end of the procedure compared to the initial mTICI (TICI shift) increased using a 3D-RA to guide MTB (*p* = 0.001) compared to an MTB performed without 3D-RA (Figure 3(b)). In addition, all patients (57/57 [100%]) in the 3D-RA group showed partial (>50%) or complete recanalization (MeVO-M-TICI 2c or 3) of the target artery compared to 27/43 (62.8%) of the control group (*p* < 0.001) (Figure 3(c)).

The number of MTB passes did not significantly differ between groups (median = 2; IQ range = 1–4, *p* = 0.411).

### Thrombectomy procedural timing

The mean overall time interval between the first 2D angiograms showing the M2 occlusion and the final control angiographic series was not significantly different (p = 0.596) between the 3D-RA group (48.7 ± 29.9 min) and control group (52.3 ± 36.5 min).

Regarding the time needed for the acquisition of the 3DRA in the 3D group, the mean time interval between the first DSA angiogram showing the M2 occlusion and the acquisition of the 3D-RA was on average 3.9 (SD ± 2.4) min. The mean interval between the 3D-RA and the acquisition of the specific 2D angiographic working projection was 4.8 (SD ± 2.0) min. Hence, the mean total time spent to acquire the specific projections since the target occlusion was detected was 8.7 (SD ± 4.4) min.

### Complications and clinical outcome

We found no significant differences in terms of good outcome (mRS ≤ 2) at 3 months between the 3D-RA group (26/53; 49.1%) and control group (18/40; 45%), as defined by the mRS (*p* = 0.834).

There was no significant difference in terms of intraprocedural complications between groups, in particular with regards to ENT and haemorrhage complications (*p* = 0.697). Regarding the clinical results at 3 months, the mRS was available for 40/43 patients of the control group and 53/57 patients for the 3D-RA group.

## Discussion

Our study shows that the guidance provided by 3D-RA to perform MTBs in patients presenting with an AIS due to a M2 occlusion allows to better target artery reperfusion compared to standard MTB conducted without such guidance. In particular 3D-RA improved the rate of good reperfusion (mTICI ≥ 2b) of the overall MCA territory compared to the control group (OR 7.1). Moreover, it increased the rate of complete target artery reperfusion (OR 7.9) and decreased the rate of residual occlusion (OR 6.8). The rate of intraprocedural complications was compared between groups and in accordance with results of other series reporting results of MTB performed in AIS patients presenting with MCA-MeVOs.^1^

Based on current treatment of cerebral aneurysms,^9^ the precise identification of the occluded artery and the vascular anatomy proximal to the occlusion are pivotal to perform an efficacious MTB.

Despite the fact that for MTBs in many cases the acquisition of oblique projections can help in the identification of the occluded branch compared to the standard AP and LL projections, it is not infrequent in our experience that oblique projections have to be corrected and reacquired more than once in order to obtain useful information regarding the point of occlusion and the vascular anatomy upstream of it. And in any case, very often the oblique projections are suboptimal compared to the 2D working projections obtained on the basis of the 3D-RA.

Prompt visualization of the occluded artery provided by the analysis of 3D-RA reconstructions, especially in the presence of multiple bifurcations or a complex vascular anatomy, together with the identification of the vessels proximal to the occlusion to be navigated to reach the clot, allows a fast and effective interaction of the thrombectomy devices with the clot.^10, 11^ Conversely, MTBs conducted under the guidance of angiograms poorly depicting the occlusion without the guidance of 3D-RA are potentially more prone to failure as thrombectomy devices could be placed in a branch not – or only partially – occupied by the clot, thus resulting in an inefficacious interaction between the devices and the clot and consequently an inefficacious clot removal.

While the use of 3D-RA reconstruction to guide the endovascular treatment of cerebral aneurysms is a well-established practice, its use is not common for MTB and, to the best of our knowledge, there are no reports on such use in the literature. One of the main reasons for which common sense precludes the use of 3D-RA in the MTB procedures is that any useless procedural steps must be avoided in order to accelerate the procedure as much as possible and recanalize the occluded artery in the shortest possible time. In our series, we found that the time spent to acquire and analyse the 3D-RA and to acquire the working projections was relatively short, on average 8.7 min. The overall MTB procedure duration was compared for both groups in our study and consistent with those reported in the literature for MCA-MeVO occlusions.^12^

The guidance provided by specific 2D-magnified working projections derived from the 3DRA, acquired with a field of view of 15 cm, allowed an easy and fast target vessel catheterization and first MTB pass. In addition, in cases of first pass failure, specific projections allowed for faster additional MTB passes, compensating for the time spent for 3D-RA acquisition and analysis.

Another reasonable concern regarding the use of 3D-RA in MTB procedures relates to the possibility that the patient will move during the procedure, thus rendering the 3D-RA unusable. In our study, all patients in the 3D-RA group were treated under general anaesthesia^13^ with appropriate blood pressure management by anaesthesiologists and were carefully curarized to prevent any slightest movement. Nevertheless, an adequate immobilization of the patient is empirically obtainable even with an efficacious conscious sedation. By contrast, the same could not be affirmed in cases of MTB performed under local anaesthesia with an uncooperative patient.

Our study has some limitations. First, we acknowledge the usual limitations related to the retrospective design of the study and to the relatively small sample size. Second, despite the higher rate of effective recanalization obtained for patients in the 3D-RA cohort, we observed no difference in terms of clinical outcome between groups. Probably one of the main reasons is that in the present study, in order to compare the technical effectiveness of thrombectomy with or without 3DRA, cases of both primary and secondary M2 occlusions were included. Therefore, the study is not powered to detect differences in clinical outcome since in primary M2 occlusions, the deficits and extensions of ischaemia are often more limited than in cases with more proximal initial occlusions, which have secondary M2 occlusions in the course of the procedure.

However, when considering only patients with primary M2 occlusions (34/57 [59.4%] patients were in the 3D-RA group and 22/43 [51.2%] in the control group), who had a distal occlusion from the beginning of the procedure, those in the 3D-RA group had a favourable outcome (mRS ≤ 2) in a higher percentage of cases than the control group (61.3% vs. 45.5%, respectively). The absence of statistical significance, despite a considerable difference in percentage, could be related to the sample size. Other factors could influence the outcome^14^ and larger, randomized populations would be needed.

Moreover, the correlation between an effective occluded vessel reperfusion obtained by MTB and improved clinical outcome has already been largely demonstrated for patients victim of an AIS secondary to large vessel occlusion.^15–17^ More recently, a number of studies evaluating the outcome of AIS patients presenting with MCA-MeVO treated by MTB also confirmed a positive correlation between reperfusion and clinical outcome^18, 19^ for this patient population. Consequently, we speculate that the guidance provided by the 3D-RA to conduct the MTB procedure can have an impact not only on the reperfusion of the occluded artery, but ultimately also on the patient's clinical outcome.

Concerning secondary M2 occlusions, despite the fact that the literature, recanalizations with final TICI 2B are considered successful, in those cases the residual occlusions are often at the level of M2 segments of branches of the MCA that irrigate important portions of the territory of the ACM (up to 50% according to the definition of TICI 2B).^20, 21^ The importance in terms of clinical outcome of trying to achieve as complete recanalizations as possible, improving the final outcome from TICI 2B to TICI 2c or 3 by means of additional distal thrombectomy maneuvers, has now been demonstrated.^19^ This becomes even more important when the occluded M2 segments belong to arteries directed to eloquent areas, such as pre- and post-central arteries.^22^

These data support the importance of implementing techniques to achieve the best possible recanalization, even in the presence of M2 segment occlusions. The use of 3DRA could become part of the armamentarium available to the neurointerventionalist, at least for the most difficult cases. In cases of occlusions distal to the M1 segment, this technique could be applied ex officio or after the failure of one or more thrombectomy maneuvers. Before discontinuing a distal thrombectomy procedure with incomplete recanalization, it might be worthwhile to perform a 3DRA and look for optimal working projections, given the relatively short time required to do so (about 9 min on average in the present study).

In principle, this concept could also apply to other MeVOs in the anterior, middle and posterior cerebral arteries.

## Conclusions

The use of 3D-RA to guide MTB allows a significantly higher reperfusion rate compared to MTB performed without 3D-RA guidance to treat AIS due to an MCA M2 segment occlusion, without loss of time. The analysis of 3D-RA reconstructions allows the prompt visualization of the occluded artery, especially in presence of a complex vascular anatomy, and results in a faster and most effective interaction of the thrombectomy devices with the clot. Further prospective, randomized trials could better investigate the value of the guidance provided by the 3D-RA for MTB in improving the quality of the occluded artery reperfusion for AIS patients presenting with MeVOs.

## Supplemental Material

**Figure:** Video 1.
